# The Cell Surface Heparan Sulfate Proteoglycan Syndecan-3 Promotes Ovarian Cancer Pathogenesis

**DOI:** 10.3390/ijms23105793

**Published:** 2022-05-21

**Authors:** Lara Hillemeyer, Nancy Adriana Espinoza-Sanchez, Burkhard Greve, Nourhan Hassan, Anca Chelariu-Raicu, Ludwig Kiesel, Martin Götte

**Affiliations:** 1 Department of Gynecology and Obstetrics, Münster University Hospital, Albert-Schweitzer-Campus 1, D11, 48149 Muenster, Germany; lara.hillemeyer@googlemail.com (L.H.); nancyadriana.espinozasanchez@ukmuenster.de (N.A.E.-S.); n_hass07@uni-muenster.de (N.H.); ludwig.kiesel@ukmuenster.de (L.K.); 2 Department of Radiotherapy-Radiooncology, University Hospital Münster, 48149 Muenster, Germany; greveb@uni-muenster.de; 3 Biotechnology/Biomolecular Chemistry Program, Faculty of Science, Cairo University, Giza 12613, Egypt; 4 Department of Obstetrics and Gynecology, University Hospital, LMU Munich, 81377 Munich, Germany; anca.chelariu-raicu@med.uni-muenchen.de

**Keywords:** heparan sulfate, proteoglycan, ovarian carcinoma, cancer stem cells, STAT3, chemotherapy, biomarker, prognosis, notch

## Abstract

Syndecans are transmembrane heparan sulfate proteoglycans that integrate signaling at the cell surface. By interacting with cytokines, signaling receptors, proteases, and extracellular matrix proteins, syndecans regulate cell proliferation, metastasis, angiogenesis, and inflammation. We analyzed public gene expression datasets to evaluate the dysregulation and potential prognostic impact of Syndecan-3 in ovarian cancer. Moreover, we performed functional in vitro analysis in syndecan-3-siRNA-treated SKOV3 and CAOV3 ovarian cancer cells. In silico analysis of public gene array datasets revealed that syndecan-3 mRNA expression was significantly increased 5.8-fold in ovarian cancer tissues (*n* = 744) and 3.4-fold in metastases (*n* = 44) compared with control tissue (*n* = 46), as independently confirmed in an RNAseq dataset on ovarian serous cystadenocarcinoma tissue (*n* = 374, controls: *n* = 133, 3.5-fold increase tumor vs. normal). Syndecan-3 siRNA knockdown impaired 3D spheroid growth and colony formation as stemness-related readouts in SKOV3 and CAOV3 cells. In SKOV3, but not in CAOV3 cells, syndecan-3 depletion reduced cell viability both under basal conditions and under chemotherapy with cisplatin, or cisplatin and paclitaxel. While analysis of the SIOVDB database did not reveal differences in Syndecan-3 expression between patients, sensitive, resistant or refractory to chemotherapy, KM Plotter analysis of 1435 ovarian cancer patients revealed that high syndecan-3 expression was associated with reduced survival in patients treated with taxol and platin. At the molecular level, a reduction in Stat3 activation and changes in the expression of Wnt and notch signaling constituents were observed. Our study suggests that up-regulation of syndecan-3 promotes the pathogenesis of ovarian cancer by modulating stemness-associated pathways.

## 1. Introduction

The syndecans are a family of four transmembrane heparan sulfate proteoglycans that are expressed in a developmental- and tissue-specific manner [1,2]. Via their heparin-related heparan sulfate carbohydrate chains, syndecans interact with a wide range of ligands that are relevant to normal cell physiology and tumor progression, including growth factors, angiogenic factors, chemokines, their respective tyrosine kinase- and G-protein coupled receptors, proteases and their inhibitors, and extracellular matrix proteins such as fibronectin, tenascin C and members of the collagen and laminin families [2,3]. Moreover, the highly conserved cytoplasmic domains of the syndecans mediate interactions with large PDZ-domain scaffolding proteins and constituents of signaling cascades [4]. Shedding—the proteolytic cleavage of the intact ectodomains of the syndecans—further expands the functional repertoire of these molecules by converting them into paracrine effector molecules [5]. Importantly, syndecans integrate a multitude of signaling pathways as cell surface coreceptors, including several pathways with oncological relevance, such as EGFR, FGFR, and IGFR signaling, the Wnt pathway, and notch signaling [6]. In this way, syndecans regulate tumor cell proliferation, apoptosis, cancer cell migration and invasion, angiogenesis, the inflammatory tumor microenvironment, and the cancer stem cell phenotype, which is associated with tumor recurrence and therapeutic resistance [1,3,6,7].

Among gynecological cancers, ovarian cancer has the highest lethality with a 5-year survival rate of 49.1% [8]. This is mainly because more than half of ovarian cancers are detected at advanced stages, for which survival is 29% in the United States and 36.30% worldwide [9] (https://gco.iarc.fr/, accessed on 24 April 2022). Even though neoadjuvant treatment followed by a less invasive surgery gained traction in the therapy of other cancers such as breast and colorectal cancers, the primary treatment of ovarian cancer is still considered radical surgery. According to the international guidelines, cytoreductive surgery in combination with platinum- and taxane-based chemotherapy represents a standard of care [10]. Over the past 10 years, several randomized phase III trials have been carried out adding bevacizumab or PARPi as maintenance therapy. These clinical studies suggested that both bevacizumab or PARPi prolong the median progression-free survival, therefore leading to multiple approvals of targeted agents in first-line maintenance [11,12,13]. However, most patients will show recurrence after first-line therapy. Moreover, despite a good clinical response to the initial treatment, the recurring tumors present substantial heterogeneity, which will lead to multiple metastases [11,14]. Therefore, given the rising development of translational research, novel approaches are needed to elucidate the molecular mechanisms and to find effective therapeutic options for patients with recurrent disease.

One potential biomarker that could be mechanistically linked to the pathogenesis of ovarian cancer is syndecan-3 (SDC3). A potential role in the pathogenesis of ovarian carcinoma was already attributed to SDC3 in 2004 [15]. In this immunohistopathological study, the expression of syndecans on the cell surface and in the extracellular matrix in the healthy ovary was compared with the expression in ovarian carcinoma. SDC3 localized to epithelial and stromal cells in tumor tissue and was also expressed in blood vessels. It showed increased expression in tumor tissue, however, no significant relationship between its expression and histological subtype or tumor grade was found [15]. A follow-up study by the authors suggested that SDC3 may be an important factor in regulating fibroblast growth factor signaling in the endothelial cells of ovarian tumor blood vessels [16]. In a study published in 2019, SDC3 was identified as a biomarker in ovarian cancer using data mining approaches including datasets of micro-dissected epithelial and stromal cells of ovarian cancers [17]. SDC3 was suggested to help distinguish benign from malignant forms of ovarian cancer. In a study, SDC3 mRNA expression was evaluated in whole blood samples from healthy donors and patients with either benign or malignant ovarian tumors. The authors found that SDC3 was one of several factors that could discriminate between benign and malignant tumors. In addition, the expression in tumor cells increased with the stage of the disease [17]. While these data point to a potential role for SDC3 in ovarian cancer pathogenesis, its full diagnostic impact and its functional relevance are not clear. In this study, we assess the expression of SDC3 in ovarian cancer tissue and metastases using large public datasets and perform functional investigations using an siRNA approach in the model cell line SKOV3.

## 2. Results

### 2.1. Syndecan-3 Is Overexpressed in Ovarian Cancer

**Figure 1:**
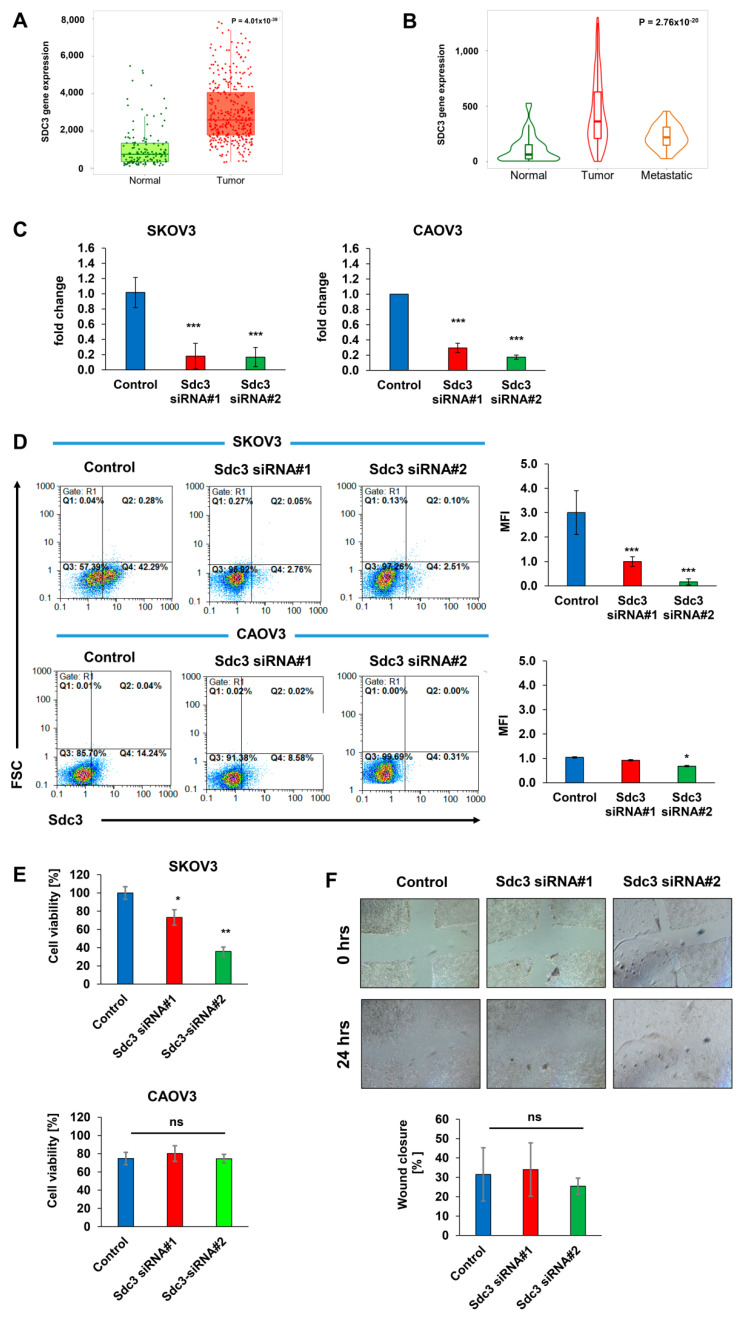
Syndecan-3 is upregulated in ovarian cancer tissues and regulates cell viability. (**A**) Syndecan-3 (SDC3) mRNA expression as analyzed by RNASeq is significantly higher in ovarian serous cystadenocarcinoma tissue (*n* = 374) compared with control tissue (*n* = 133), *p* = 4.01 × 10^−39^, Mann–Whitney test; (**B**) SDC3 mRNA expression as determined by gene array analysis is significantly higher in ovarian cancer (*n* = 744) and ovarian cancer metastatic tissue (*n* = 44) compared with control tissue (*n* = 46), *p* = 2.76 × 10^−20^, Kruskal–Wallis test; *p* = 1.27 × 10^−17^ (normal vs. tumor), *p* = 9.43 × 10^−7^ (normal vs. metastasis), *p* = 4.75 × 10^−3^ (tumor vs. metastasis), Dunn’s test; data shown in (**A**,**B**) were retrieved from the TNMPlot.com database (access date: 4 June 2021) [18], which combines public gene expression data on ovarian cancer, ovarian cancer metastases and normal tissue from the GEO, GTEx and TCGA datasets. (**C**) SDC3 siRNA #1 and #2 knockdown in human SKOV3 and CAOV3 ovarian cancer cells results in a >80% downregulation of SDC3 mRNA expression in SKOV3 cells compared with control siRNA-treated cells while in CAOV3 cells, >70% and >80% downregulation of SDC3 expression was observed when the cells were treated with the siRNA#1 and siRNA#2, respectively, as determined by qPCR. *n* = 5, *** = *p* < 0.001, error bars = SD. (**D**) SDC3 siRNA knockdown in human SKOV3 and CAOV3 ovarian cancer cells results in a >67% and a 10% downregulation of Sdc3 cell surface protein levels compared with control siRNA-treated cells with siRNA #1, respectively. With siRNA#2 a >90% and a 30% downregulation of SDC3 in SKOV3 and CAOV3 is observed, respectively, as determined by flow cytometry. Representative dot plots are shown. MFI = mean fluorescence intensity, *n* = 3, *** = *p* < 0.001 and * = *p* < 0.05 error bars = SD. (**E**) SDC3 siRNA knockdown reduces SKOV3 cell viability by 30% with siRNA#1 and by 70% with siRNA#2 compared with control siRNA-treated cells, as determined by MTT assay. *n* = 4, * = *p* < 0.05, ** *p* < 0.01, error bars = SD; (**F**) SDC3 siRNA depletion does not affect SKOV3 cell motility as determined by scratch wound assay. SKOV3 cells were transfected with either control siRNA or SDC3 siRNA#1 and #2 and subjected to a 24 h scratch wound assay 72 h after transfection. Shown is the percent reduction of the cell-free area at the endpoint (*t* = 24 h) compared with the original wounded area at *t* = 0 h. *n* = 5; *p* > 0.05 (ns = not significant), error bars = SD.

To assess the potential clinicopathological relevance of SDC3 expression in ovarian cancer, we analyzed its expression in tumor and control tissues using the online tool TNMplot (https://www.tnmplot.com, accessed on 4 June 2021) [18], which combines public gene expression data on ovarian cancer specimens, ovarian cancer metastases and normal tissue from the GEO, GTEx and TCGA datasets. Analysis of RNASeq data revealed that SDC3 mRNA expression is significantly upregulated 3.5-fold in ovarian serous cystadenocarcinoma tissue (*n* = 374) compared with control tissue (*n* = 133) (*p* = 4.01 × 10^−39^) (Figure 1A). Independent analysis of Affymetrix gene expression datasets in ovarian cancer tissue (*n* = 744), ovarian cancer metastases (*n* = 44), and normal tissue (*n* = 46) confirmed a significant upregulation of SDC3 in tumor tissue (5.8-fold, *p* = 1.27 × 10^−17^) and metastases (3.4-fold, *p* = 9.43 × 10^−7^) compared with normal tissue. Notably, SDC3 expression was 1.7-fold lower in metastases compared with primary tumors (*p* = 4.75 × 10^−3^), suggesting possible differential roles for SDC3 in the early and late stages of tumorigenesis (Figure 1B).

### 2.2. Syndecan-3 Depletion Affects Colony Formation and 3D Spheroid Growth of Human SKOV3 and CAOV3 Ovarian Cancer Cells

Since the significant upregulation of SDC3 in ovarian cancer tissues suggested a possible role in the pathogenesis of the disease, we studied the impact of SDC3 downregulation in vitro using a siRNA knockdown approach in SKOV3 and CAOV3 cells, two widely used model cell lines of human ovarian carcinoma [19]. Both cell lines present epithelial morphology and are derived from ovarian tumors, however, SKOV3 is derived from a non-serous carcinoma, while CAOV3 is derived from high-grade serous ovarian cancer. We employed two independent SDC3 siRNA sequences, targeting exon 5 (siRNA#1) and exon 3 (siRNA#2) of human SDC3 mRNA (NM_014654.3). We observed >80% downregulation of SDC3 expression in SKOV3 cells at the mRNA level with both siRNAs compared with control siRNA-treated cells as determined by qPCR (Figure 1C, left panel). Furthermore, flow cytometry revealed a >90% downregulation of Sdc3 protein expression at the surface of SDC3-depleted SKOV3 cells with both siRNAs compared with controls (Figure 1D, upper panel). In CAOV3 cells, we found a >70% downregulation in the expression of SDC3 with siRNA#1 and a >80% downregulation with siRNA#2 (Figure 1C, right panel). The flow cytometry analysis reveals that the downregulation of SDC3 with siRNA#1 resulted in 40% fewer SDC3-positive cells, confirming the impact of SDC3 depletion at the protein level. However, among a pool of remaining SDC3-positive cells, the MFI was not reduced. In cells treated with the SDC3 siRNA#2, we observed 90% fewer SDC3-positive cells and a significant reduction in the MFI (Figure 1D, bottom panel).

We next analyzed the impact of SDC3 siRNA knockdown on cell viability using the metabolic MTT assay. SDC3 depletion significantly reduced SKOV3 cell viability by 30% (siRNA#1) and 65% (siRNA#2 (Figure 1E), while the viability of CAOV3 cells was not affected after transfection with the two types of siRNAs (Figure 1E). To assess the potential role of SDC3 in regulating cell motility, we performed scratch wound closure assays. SDC3 depletion did not result in altered cell migration both in the presence of serum (Figure 1F) and under serum-depleted conditions (results not shown). Since we did not observe any changes in SKOV3 cell migration, we decided not to perform this experiment on CAOV3 cells.

**Figure 2:**
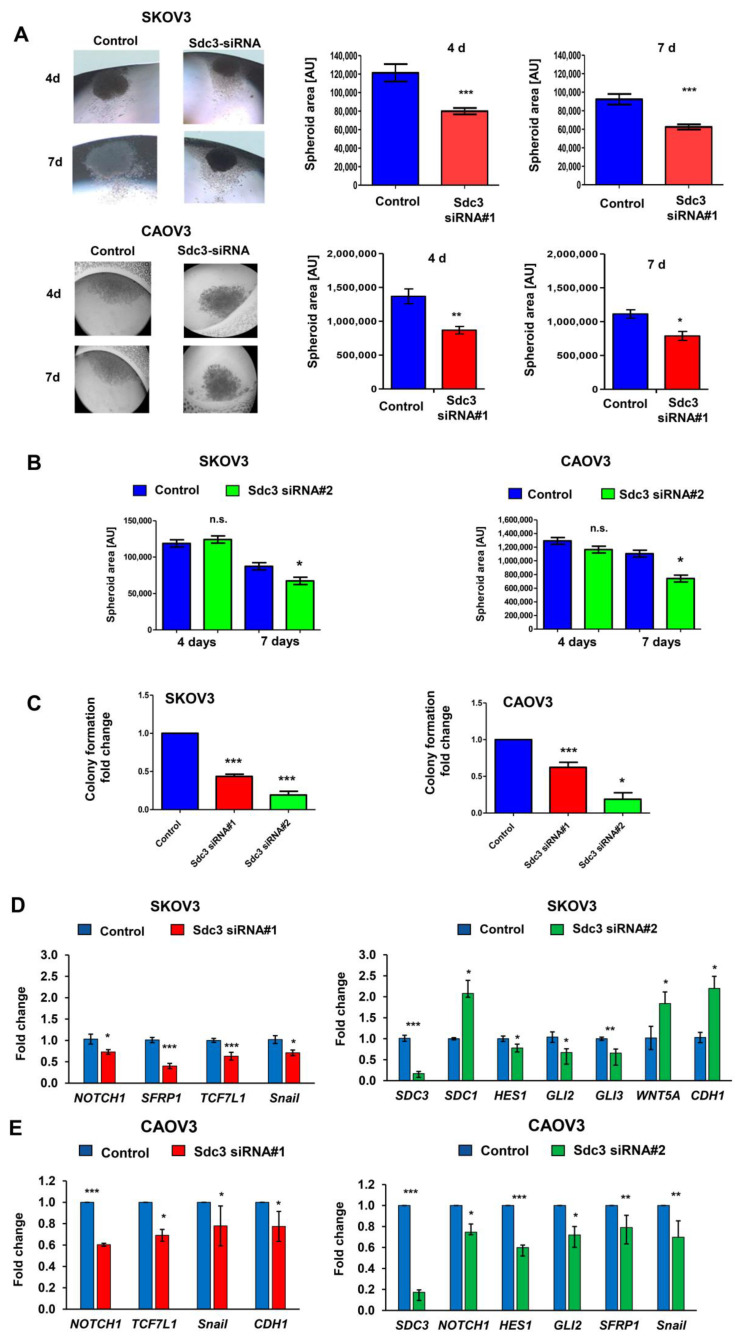
SDC3 depletion reduces the size of 3D SKOV3 spheroids and alters stemness-related gene expression. (**A**) Control siRNA and SDC3-siRNA#1- and #2-treated SKOV3 and CAOV3 cells were subjected to a hanging drop assay, allowing for the formation of 3D spheroids [24]. Representative pictures of the hanging drop cultures are presented in the left panels and quantitative analysis in the right panels. The spheroid area was quantified using NIH Image J software. In SDC3-siRNA#1-treated SKOV3 and CAOV3 cells, the spheroid area was significantly reduced after 4 days (*p* < 0.001, *n* = 4, error bars = SEM) and 7 days (*p* < 0.001, *n* = 4, error bars = SEM) of hanging drop culture, respectively. (**B**) Quantitative analysis of the spheroid area in SDC3-siRNA#2-treated SKOV3 and CAOV3 cells. The spheroid area was significantly reduced after 7 days (* *p* < 0.05, error bars = SEM). (**C**) Colony formation was decreased in SDC3-siRNA#1- and #2-treated SKOV3 and CAOV3 cells. *** = *p* < 0.001 and * = *p* < 0.05 error bars = SD. (**D**,**E**) qPCR analysis reveals significant mRNA expression changes of constituents of the stemness-related notch and Wnt signaling pathways and of the EMT marker Snail in SKOV3 (**D**) and CAOV3 (**E**) after knockdown of SDC3 with the two siRNAs. *** = *p* < 0.001, ** = *p* < 0.01, * = *p* < 0.05, *n* = 4, error bars = SEM.

Since we previously showed that heparan sulfate proteoglycans are capable of modulating breast and colon cancer stem cell properties, associated with therapeutic resistance [20,21,22,23], we evaluated the potential influence of SDC3 on the growth of three-dimensional spheroids as a readout of stem cell activity in a hanging drop assay [24]. Both SDC3-depleted and control siRNA-treated SKOV3 and CAOV3 cells formed dense spheroids after 4 days of culture that were surrounded by a margin of more loosely arranged cells (Figure 2A, left panel). Quantification of the dense core area revealed that SDC3 depletion resulted in a significant decrease in sphere size by >30%, which was particularly apparent after 7 days of culture in both cell lines (Figure 2A, right panel). We performed the same analysis with the second SDC3 siRNA and interestingly, we observed a reduction in the spheroid area only after 7 days in both cell lines (Figure 2B). To corroborate these results, we performed colony formation assays in SKOV3 and CAOV3 cells using the two SDC3 siRNAs. We observed 50% less capacity for colony formation in both cell lines after the knockdown of SDC3 with siRNA#1 (Figure 2C). The inhibition of the capacity of colony formation was even stronger using siRNA#2 (Figure 2C). In addition, we observed a clear difference in the morphology of the colonies formed by both cell lines. While SKOV3 cells form larger and undefined colonies, CAOV3 cells form much smaller and more compact colonies (Supplementary Figure S2).

Since heparan sulfate proteoglycans modulate numerous signaling pathways relevant to cancer stem cell properties [7,25], we next used qPCR analysis to investigate the impact of SDC3 depletion on the expression of constituents of the notch pathway (*Notch1*, *Hes1*, *Hey2*, *Msi1*, *Msi2*), hedgehog signaling (*SHH*, *PTCH1*, *Gli1-3*, *Robo1*), Wnt signaling (*Wnt3A*, *Wnt 5A*, *SFRP1*, *TCF7L1*, *TCF7L2*), and markers of epithelial-to-mesenchymal transition (EMT) (*Twist*, *Snail*, *CDH1*, *Vimentin*, *ZEB1*) by SKOV3 and CAOV3 cells. Moreover, we evaluated the expression of the other members of the syndecan family (*SDC1*, *SDC2*, *SDC4*) for potential compensatory expression changes, and assessed the expression of the proto-oncogene tyrosine-protein kinase Src, previously described to interact with Sdc3 [26]. In SKOV3 cells, no significant SDC3-dependent changes in gene expression were observed for *Hey2*, *Msi1*, *Msi2*, *SHH*, *PTCH1*, *Robo1*, *Wnt3A*, *TCF7L2*, *Twist*, *Vimentin*, *ZEB1*, *SDC1*, *SDC2*, *SDC4* and *Src* (Supplementary Figure S1A). A non-significant trend for a differential regulation was observed for the hedgehog pathway constituents *Gli1* and *Gli3*, along with a modest effect on the epithelial marker E-cadherin (*CDH1*) (Supplementary Figure S1A). Syndecan-3 depletion by SDC3 siRNA#1 resulted in a significant downregulation of *Notch1*, the Wnt pathway constituents *SFRP*1 and *TCF7L1*, and the mesenchymal marker *Snail* (Figure 2D, left panel). When SDC3 was knocked down using siRNA#2, downregulation of the notch-dependent transcription factor *Hes1* and the hedgehog-dependent transcription factors *Gli2* and *Gli3* was observed (Figure 2D, right panel). In contrast, the Wnt pathway ligand *Wnt5A*, and the epithelial marker *E-cadherin* were significantly upregulated in SDC3-depleted cells (Figure 2D). Notably, in contrast to SDC3 siRNA#1, siRNA#2 induced a significant, potentially compensatory upregulation of SDC1 expression, which may have contributed to the observed differences (Figure 2D) (see discussion). In CAOV3 cells, with SDC3 depletion by SDC3 siRNA#1 we observed a significant downregulation of *Notch1*, *TCF7L1*, and the mesenchymal markers *Snail* and *E-cadherin* (*CDH1*) (Figure 2E, left panel). siRNA knockdown using SDC3 siRNA#2 resulted in downregulation of *Notch1*, *Hes1*, *Gli2*, *SFRP1*, and *E-cadherin* in CAOV3 cells (Figure 2E, right panel). We did not observe significant differences in the expression of the other genes analyzed (Supplementary Figure S1B). While some cell-type-specific differences were observed (see discussion), we conclude that SDC3 depletion in SKOV3 and CAOV3 ovarian cancer cells resulted in a downregulation of stemness-related constituents of the Notch, Wnt, and hedgehog pathways.

### 2.3. Impact of Syndecan-3 on the Chemotherapy Response in Ovarian Cancer

**Figure 3:**
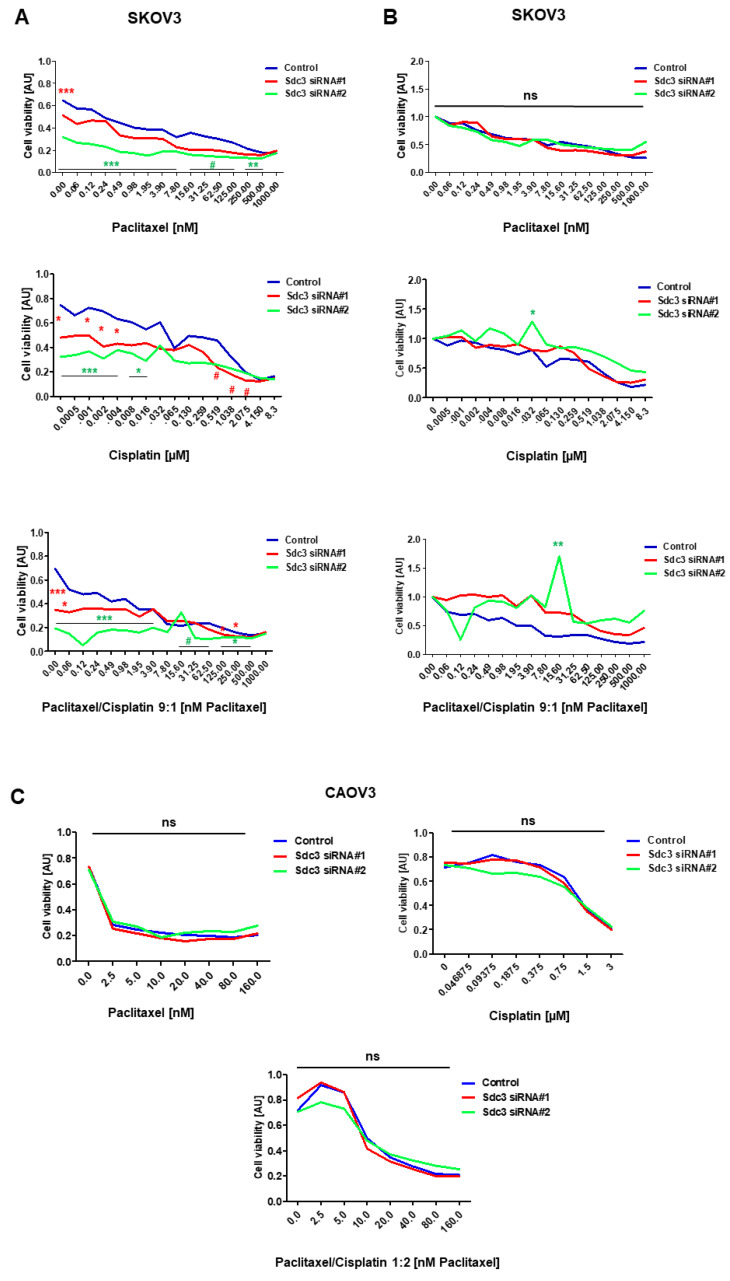
In vitro investigation of the chemotherapy response in SDC3-depleted ovarian cancer cell lines. (**A**) Response of SDC3 siRNA and control siRNA#1- and #2-treated SKOV3 cells subjected to in vitro treatment with paclitaxel (0–1000 nM), cisplatin (0–8.3 µM) or combinatorial treatment (9:1 paclitaxel/cisplatin (0–1000 nM paclitaxel, 0–114 nM cisplatin) for 72 h. Cell viability was measured by the MTT assay. SDC3-depleted cells showed reduced basal cell viability and under treatment with cisplatin and combined paclitaxel/cisplatin treatment at the indicated concentrations. (**B**) Response of SDC3 siRNA control and siRNA#1- and #2-treated SKOV3 cells after the normalization against the untreated cells of each condition. (**C**) Response of SDC3 siRNA and control siRNA#1- and #2-treated SKOV3 cells subjected to in vitro treatment with paclitaxel (0–160 nM), cisplatin (0–3 µM) or combinatorial treatment (9:1 paclitaxel/cisplatin (0–1000 nM paclitaxel, 0–3 µM cisplatin) for 72 h. Cell viability was measured by the MTT assay. SDC3-depleted cells did not show differences in basal cell viability and under treatment with cisplatin and combined paclitaxel/cisplatin treatment at the indicated concentrations. *** = *p* < 0.001, ** = *p* < 0.01, * = *p* < 0.05, # = *p* < 0.08 (non-significant trend), ns = not significant; *n* = 8; blue (control), red SDC3 knockdown siRNA#1 and green SDC3 knockdown siRNA#2 lines represent mean values.

Cancer stem cell properties have been linked to cancer recurrence and resistance to chemotherapy due to the high expression of multidrug resistance proteins [7]. As our results indicated an impact of SDC3 on cancer-stem-cell-related gene expression patterns and functional properties, we next evaluated the response of SDC3-depleted and control SKOV3 and CAOV3 cells to the chemotherapeutics paclitaxel, cisplatin, and a combinatorial treatment with these drugs in vitro. In MTT assays, SDC3-depleted SKOV3 cells showed significantly less viability under baseline conditions and when treated with cisplatin or a combination of cisplatin and paclitaxel over a wide range of concentrations (Figure 3A). However, upon normalization to the untreated control condition, this effect appeared to be due to the impact of Sdc3 on cell viability rather than an effect on the chemotherapy response (Figure 3B). We only observed a significant difference in cells treated with siRNA#2 at the 0.032 µM dose of cisplatin and the 15.60 nM dose of paclitaxel/cisplatin (Figure 3B). While in CAOV3 cells treated with either SDC3 siRNA#1 or siRNA#2, concerning the control, we observed no significant difference (Figure 3C). The IC_50_ for each drug and treatment as well as the combination index can be found in Table 1. In conclusion, we observed that in SKOV3 cells, SDC3 expression has an impact on cell viability, whereas no direct effect on the therapy response was observed in either cell line.

**Figure 4:**
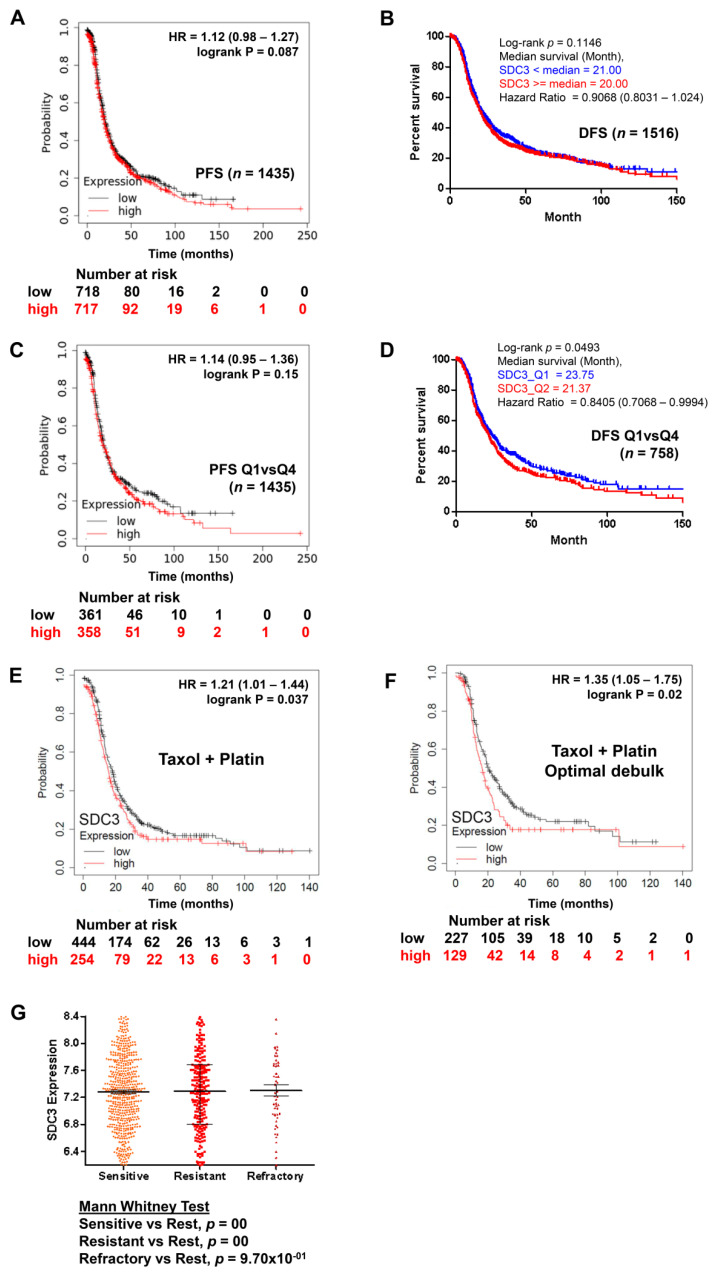
Kaplan–Meier survival analysis. (**A**) all patients, (**C**) Quartile 1 and Quartile 4 expression of SDC3 in ovarian cancer patients. (**E**) Ovarian cancer patients with high compared with low SDC3 mRNA expression in tumor tissue in patients subjected to Taxol and platinum chemotherapy (*p* = 0.037, hazard ratio (HR) = 1.21) showed a worse survival of (**F**) shows the patient subgroup with Taxol and platinum chemotherapy and optimal debulking (*p* = 0.02, HR = 1.35). (**A**,**C**,**E**,**F**) Data were retrieved from the KM Plotter database [27], which combines public gene expression data based on Affymetrix gene array analyses of 1435 ovarian cancer patients in the GEO and TCGA datasets. The default threshold settings of the KM Plotter tool and a 180-month follow-up were selected for the analysis. (**B**,**D**,**G**) Data were retrieved from the CSIOVDB database [28], retrieved on 26 March 2022. (**B**) All patients, (**D**) Quartile 1 (blue) and Quartile 4 (red) expression of SDC3 in ovarian cancer patients. (**G**) SDC3 expression and chemotherapy response. Mann–Whitney test was performed to evaluate statistical significance.

Subsequently, we evaluated the impact of SDC3 expression on the prognosis of ovarian cancer patients overall, by quartile expression, and subjected to chemotherapy, using the public online resource KM plotter, which combines public gene expression data based on Affymetrix gene array analyses of 1435 ovarian cancer patients in the GEO and TCGA datasets [27] and using and the CSIOVDB platform which provides integration with the copy number, DNA methylation, and mutation data from TCGA [28]. No significant correlation was observed between SDC3 expression and the survival of patients overall, in both KMplot and CSIOVDB analysis (Figure 4A,B). When the patients were classified by quartiles, only in the analysis with the CSIOVDB platform did SDC3 correlate with better disease-free survival (*n* = 758, *p* = 0.0493) (Figure 4C,D) and overall survival (*n* = 934, *p* = 0.01439). Using the default threshold settings and a follow-up of 180 months in KMplot analysis, patients receiving combinatorial chemotherapy with Taxol and cisplatin showed a significantly reduced progression-free survival when SDC3 expression was high (*n* = 254, median survival 15.63 months), compared with patients with low tumoral SDC3 expression (*n* = 444, median survival 18 months, *p* = 0.037, hazard ratio (HR) = 1.21) (Figure 4E). In the subgroup of patients with optimal debulking and combinatorial Taxol/platinum chemotherapy, the results were even more clear, with a median survival of 16.37 months in the SDC3-high subgroup (*n* = 129) and 20 months in the SDC3-low subgroup (*n* = 227; *p* = 0.02, HR = 1.35) (Figure 4F). In the CSIOVDB analysis, no differences in the mean SDC3 expression were observed between patients with a sensitive, resistant, or refractory therapy response (Figure 4G). However, in these patients, it is not defined what type of treatment they received, while in the KMplot analysis the patients were specifically analyzed for response to therapy with Taxol and cisplatin. In conclusion, although Kmplot analysis shows that in some patients SDC3 expression has a negative impact on their survival depending on the treatment they had, in vitro studies as well as analysis using CSIOVDB show no correlation between SDC expression, chemotherapy resistance, and survival. We can only speculate if the improved prognostic value observed in the Kmplot subgroup analysis may originate from the more homogenous group of patients with platin–taxane treatment and optimal debulking. Therefore, due to the basal effect on cell viability, it is not clear at this point whether SDC3 directly influences the response to therapy and in consequence, the survival of the patients, and further studies are needed to independently confirm if its expression is linked to patient survival in specific subgroups.

### 2.4. Syndecan-3 Shows Functional Interactions with Stat3 Signaling and Is Associated with Stemness-Associated Signaling Pathways in STRING Analysis

**Figure 5:**
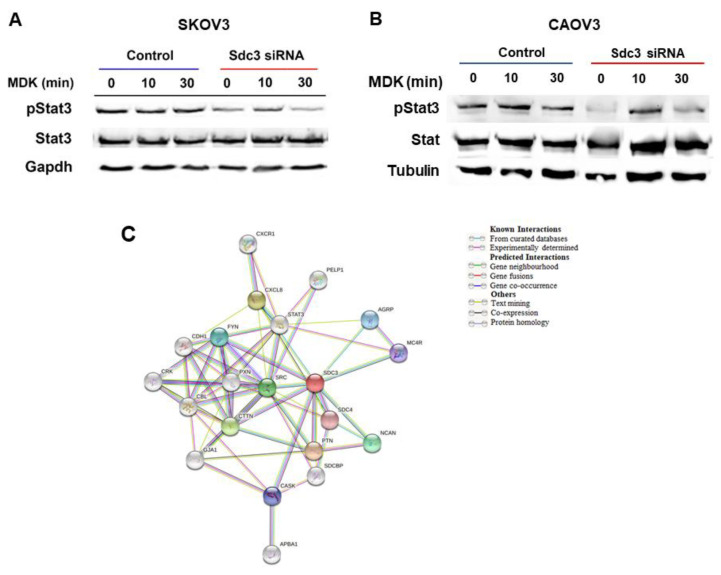
SDC3 siRNA#1 depletion in (**A**) SKOV3 and (**B**) CAOV3 cells results in reduced activation of Stat3. SKOV3 and CAOV3 cells were subjected to control or SDC3 siRNA#1 treatment, serum starved for 24 h and stimulated with 2 ng/mL of the cytokine Midkine (MDK) for the indicated timepoints. Cell lysates were subjected to Western blotting for the active, phosphorylated form of Stat3, total Stat3 and Gapdh as a loading control. SDC3 depletion reduces the constitutive activation of Stat3 in both cell lines. Representative blot from 3 experiments. Total Stat3 and Gapdh were detected on the same membrane after antibody stripping. (**C**) String analysis reveals direct functional interactions of SDC3 with SDC1 and Notch1 and indirect interactions with constituents of the stemness-associated Notch, Wnt and hedgehog pathways. Interaction analysis of SDC3 with SDC1, STAT3, the Notch pathway constituents NOTCH1 and HES1, the Wnt signaling constituents WNT5A and TCF7L1, the hedgehog pathway constituents GLI1–3, and the EMT-related markers E-cadherin (CDH1) and Snail (SNAI1) was performed using the STRING database (http://string-db.org/, accessed on 4 June 2021).

To further evaluate possible mechanisms by which SDC3 modulates cell viability and stemness-associated factors in SKOV3 and CAOV3 cells, we investigated signaling constituents that have been previously linked to SDC3 function. For example, Sdc3 has been shown to interact with the cytokine Midkine [29] and with the Src proto-oncogene [26] in a neurobiological context. We therefore investigated the response of control and SDC3-siRNA-treated SKOV3 and CAOV3 cells to Midkine stimulation using the phosphorylation of the Stat3 transcription factor as a readout [30]. Western blot analysis revealed that Midkine did not increase Stat3 phosphorylation in serum-starved control cells, possibly due to the presence of constitutive Stat3 activation already in unstimulated cells in both cell lines (Figure 5A,B). The cell viability of SKOV3 and CAOV3 cells was not affected by Midkine stimulation (results not shown). In contrast to control siRNA-treated cells, SDC3-depleted cells showed lower activation levels of Stat3 (Figure 5A,B). When the expression of Src was investigated by Western blotting of SDC3-depleted and control SKOV3 and CAOV3 cell extracts, no consistent changes in its expression were observed (results not shown). To further characterize molecular interactions of SDC3, we performed an unbiased in silico analysis using the STRING (Search Tool for the Retrieval of Interacting Genes/Proteins) online tool [31] (Figure 5C). The analysis demonstrated that SDC3 interacts directly with proteins belonging to signaling pathways such as Src, Fyn, and Stat3 and interacts indirectly with chemokines such as Cxcl8 and Cxcr1 through Src and Stat3. Notably, of the genes for which we found a differential expression after SDC3 knockdown, Cdh1 is indirectly linked to SDC3 through different factors such as Src, Cxcl8, and Fyn. As we expected, there is also a direct association between SDC3 and other matrix proteins such as SDC4 and NCAN. Interestingly, SDC3 also interacts with proteins associated with homeostasis and development such as Agrp, Mc4r, and Cask.

## 3. Discussion

Previous studies on the expression of SDC3 in ovarian cancer had pointed to its misexpression in diseased tissue and possible utility as a biomarker, however, the reported data fell short of significance and revealed only trends [15,17]. Through the in silico analysis of large public gene expression datasets, we were now able to demonstrate that SDC3 mRNA expression is highly significantly upregulated in ovarian cancer tissue in comparison with control tissue. Moreover, SDC3 was more highly expressed in metastases compared to healthy tissue, however, expression in metastases was lower than in primary tumors. We can only speculate on the underlying reasons for this finding, which could suggest that SDC3 may play a more important role in tumor progression at the early stages of the disease compared with later stages. Also in melanoma, Sdc3 expression is induced by hypoxia-inducible factors such as HIF1α. Interestingly, cells of the tumor microenvironment such as macrophages and endothelial cells express Sdc3 [32]. In pancreatic ductal carcinoma, Sdc3 expression correlates with primary tumor size in a mouse model [33]. This suggests that Sdc3 expression by both tumor cells and the microenvironment have an impact on tumor progression.

Our in vitro findings are in line with this hypothesis, as SDC3 knockdown in ovarian cancer cells primarily affected cell viability (as a readout of tumor growth), but not cell motility as one of the prerequisites for metastatic spread. Nevertheless, the lack of an impact of SDC3 depletion in the wound repair assays is surprising, as a role for SDC3 in cell motility has been previously suggested in a neurobiological and inflammatory context [34,35]. As syndecans act as multifunctional integrators of signal transduction at the cell surface [6], the negative impact of SDC3 depletion on cell viability may have been due to a dampening of such signaling events. In vitro, stimulation with the cytokine, Midkine, previously suggested to interact with SDC3 in other experimental systems [29,36,37], did not enhance Stat3 signaling or cell viability in control SKOV3 or CAOV3 cells. We ascribe this to the constitutive activation of Stat3 signaling, and possibly other signaling pathways in these cells, as phosphorylated Stat3 was already seen in the absence of the Midkine stimulus in unstimulated cells. Indeed, while we could not detect expression changes of Src, a proto-oncogene previously linked to SDC3 function [26], activation of the inflammation- and stemness-related transcriptional regulator Stat3 was reduced in SDC3-depleted SKOV3 and CAOV3 cells, by the classical co-receptor role for syndecans in cytokine signaling [38,39,40,41].

Apart from its role in inflammation, Stat3 signaling has also been linked to a cancer stem cell phenotype in the context of another syndecan member, syndecan-1, and to a cancer stem cell phenotype and resistance to chemotherapy in ovarian cancer [39,42,43,44,45]. Therefore, reduced Stat3 signaling in SDC3-depleted cells may be linked to the reduction in both cell lines’ 3D spheroid growth as a readout of putative stem cell function. Indeed, SDC3 has well-documented roles in physiological stem cell function, including bone-marrow-derived mesenchymal stem cells [40], satellite cells in the context of myogenesis [46,47], and possible roles in neuronal development [48,49]. Our qPCR analysis revealed that several constituents of stemness-related signaling pathways were dysregulated upon SDC3 depletion in ovarian cancer cells in vitro, the most notable constituents of the Notch, Wnt and hedgehog pathways. Our STRING analysis demonstrates the interdependences of these pathways and links them to the signaling factor Stat3. In line with our analysis, various studies show the correlation between heparan sulfate proteoglycans and stemness-related signaling [7,22,39,50]. For example, increased 3-O-sulfation of heparan sulfate enhances expression of the Wnt-dependent transcription factor TFF4/TCF7L2 and 3D sphere formation in breast cancer cells and results in differential expression of constituents of the prognostically relevant hedgehog pathway [21,25,51]. Altered 3-o-sulfation of heparan sulfate has also been shown to sensitize ovarian carcinoma to oncogenic signals and to be a predictor of prognosis [50]. Proteoglycans also play a role in the activation of the Notch signaling pathway, as sulphated proteoglycan chains activate Notch in the fruit fly *Drosophila* [52]. Indeed, SDC3 and notch cooperate during adult myogenesis [46], and similar mechanisms could be active in our experimental system.

The impact of SDC3 on stemness-associated gene expression patterns and 3D spheroid formation may be functionally linked to resistance to chemotherapy in ovarian cancer. Indeed, due to a higher expression of multidrug resistance proteins, dedicated detoxification systems, and a dormant, non-mitotically active state, so-called cancer stem cells are thought to be particularly therapy resistant, representing a subpopulation within tumors that are associated with recurrence [7,53,54,55]. Consistent with this concept, our KM Plotter analysis revealed that a high SDC3 expression was associated with worse survival in ovarian cancer patients treated with Taxol and platin chemotherapy, indicating a possible functional role for SDC3 in resistance to chemotherapy. While SDC3-depleted SKOV3 cells showed reduced viability under combinatorial treatment with paclitaxel and cisplatin, and under cisplatin monotherapy over a wide range of concentrations in vitro, this effect was confounded by the SDC3-dependent reduction of cell viability already at untreated baseline levels. Moreover, no effect on chemotherapy resistance and baseline cell viability was seen upon SDC3 depletion in CAOV3 cells, and analysis of the CSIOVDB database did not show changes in the mean expression levels of SDC3 in tumors of patients sensitive, resistant or refractory to chemotherapy. Therefore, our data may point to a potential role for SDC3 in chemoresistance in a subgroup of ovarian cancer patients rather than a general link to therapeutic resistance in ovarian cancer.

The resistance of cancer cells to chemotherapeutic agents is based on various mechanisms such as p53, gene mutations, and dysfunctional DNA repair mechanisms [56,57,58]. In addition, as mentioned above, cancer stem cells [7,52,53,54] and epithelial–mesenchymal transition (EMT) [59] are associated with resistance. Indeed, genes of the hedgehog pathway, found to be affected by SDC3 knockdown in this study, were also expressed more frequently in cancer stem cells [53]. EMT is a process where cells change from an epithelial phenotype to a mesenchymal phenotype. During this transition, the cells lose basal polarity and cell–cell adhesion and become invasive mesenchymal cells. Many epithelial-derived ovarian cancer cells show a mesenchymal phenotype, especially platinum-resistant cells [60]. These pathways lead to the activation of EMT transcription factors. For example, when a ligand binds to the PTCH1 receptor of the sonic hedgehog pathway, the SMO receptor is activated, which in turn activates Gli transcription factors, causing increased Snail expression [61]. Snail can decrease the expression of E-cadherin, which enables the conversion of epithelial to mesenchymal cells [62]. Moreover, non-canonical WNT5a was shown to regulate EMT in the mouse ovarian surface epithelium, with possible relevance for our experimental system [63]. Indeed, Wnt5a and other constituents of the Wnt pathway have been suggested as promising therapeutic targets in ovarian cancer, as activation of this pathway has been linked to the progression of the disease [64,65]. Finally, upstream regulators such as autocrine relaxin signaling may represent attractive therapeutic targets in ovarian cancer, as they drive the expression of Wnt and Notch pathway constituents [66]. Our study suggests that further evaluation of SDC3 in this context may expand the repertoire of therapeutic targets. Therapeutic monoclonal antibodies, CAR-T-NK cells, vaccines, and immunotoxins to target proteoglycans are currently under development and have shown promising preclinical effects [3].

A few caveats are associated with our study. Regarding the survival analysis, a significant impact of SDC3 expression on survival could only be demonstrated for the combinatorial treatment with Taxol and platin, but not in other settings. Differences in patient populations in the public databases that we employed could explain some of the differences in our clinical datasets. For example, CSIOVDB emphasizes that it does not mirror the actual frequency of ovarian cancer, where non-epithelial ovarian cancer accounts for 10% of all ovarian cancers [28]. Moreover, while both SDC3 siRNA sequences affected the same stemness-associated pathways, individual differences in the affected genes were noted. We ascribe this to the possibly compensatory upregulation of SDC1 expression in SKOV3 cells treated with SDC3 siRNA#2, which may have acted as a confounder. The upregulation of E-cadherin in these cells may also be linked to this phenomenon, as we and others have previously observed co-regulation of E-cadherin and SDC1 in other experimental systems [67,68]. Moreover, differences in knockdown efficiency of the two siRNA sequences may have had an impact on the regulation of individual constituents of these pathways. We want to emphasize that both SDC3 siRNA sequences produced consistent results in all functional assays except for CAOV3 cells in the MTT assays, and were consistent in the pathways regulated by SDC3 knockdown in vitro.

In summary, our study has demonstrated that SDC3 is over-expressed in ovarian cancer and that its upregulation is associated with a poor prognosis in the subgroup of patients receiving Taxol and cisplatin chemotherapy. At the functional level, SDC3 depletion had an impact on colony formation and 3D spheroid formation as readouts of putative stem cell activity, which was associated with reduced expression of constituents of the stemness-associated Notch, hedgehog, and Wnt pathways and reduced Stat3 activation. Therefore, the future evaluation of SDC3 as a therapeutic target in advanced preclinical models appears worthwhile.

## 4. Materials and Methods

### 4.1. Cell Culture

The human ovarian cancer cell lines SKOV3 and CAOV3 were purchased from ATCC/LGC Promochem (Wesel, Germany). SKOV3 cells were cultured in McCoys 5A medium containing 10% fetal calf serum (FCS) (Biochrom GmbH, cat. no. S0615, Berlin, Germany), and 1% penicillin/streptomycin (cat. no. P4333) and CaOV3 cells were cultured in RPMI medium containing 10% FCS and 1% penicillin/streptomycin in a humidified atmosphere of 7.5% CO_2_ for SKOV3 and 5% CO_2_ for CAOV3 at 37 °C. All reagents except for FCS were purchased from Sigma-Aldrich Chemie GmbH (Taufkirchen, Germany).

### 4.2. siRNA Transfection

SKOV3 and CaOV3 cells were cultured in growth media at a density of 3 × 10^3^ cells/well of a six-well plate for 24 h. After 24 h, siRNA transfection was performed using 20 nM silencer pre-validated siRNA (Ambion, cat. No. #s18614 designated as SDC3 siRNA#1, targeting exon 5 (NM_014654.3), and #s228527 designated as SDC3 siRNA#2, targeting exon 3 (NM_014654.3), Cambridgeshire, UK) to silence SDC3, or a silencer™ select negative control siRNA (Ambion, Austin, TX, USA, cat. No. 4390844) using Dharmafect reagent (Dharmacon™, cat. No. T-2001-03, Lafayette, CO, USA) according to the manufacturer’s protocol. This reagent contained in a total volume of 1 mL, 840 μL Opti-MEM^®^ media (Gibco^®^, Thermo-scientific, Dreieich, Germany), 80 μL 20 nM Sdc3 siRNA#1 and #2/Opti-MEM^®^ or negative control siRNA, and 80 μL 2.5% Dharmafect/Opti-MEM^®^ solution. The knockdown efficiency was confirmed by RT-qPCR and flow cytometry.

### 4.3. Total RNA Extraction and cDNA Synthesis

Total RNA extraction from silenced SKOV3 and CaOV3 cells was performed 72 h after transfection with the InnuPREP RNA mini kit (Analytikjena, cat. no. 845-KS-2040250, Jena, Germany) following the manufacturer’s instructions. Reverse transcription of mRNA (from 1 µg of total RNA) into cDNA was performed with the High-Capacity cDNA Reverse Transcription Kit (Applied Biosystems, cat. no. 4368814, Foster City, CA, USA).

### 4.4. Quantitative Real-Time PCR

Quantitative real-time PCR (qPCR) for both cell lines was performed, RT2 SYBR Green qPCR Primer Assay (Qiagen, cat. no. 330500, Hilden, Germany) and Takyon™ ROX probe qPCR Kit (Eurogentec GmbH, cat. no. UF-RPMT-B0100, Cologne, Germany) in a 7300 real-time PCR detection system (Applied Biosystems, Darmstadt, Germany). The relative gene expression levels were assessed using the 2−ΔΔCt method after normalization to GAPDH gene expression as an internal control. Melting curve analysis was conducted to confirm specific product amplification. Gene expression of SDC1, SDC2, and SDC4 was analyzed using the TaqMan probes Hs00174579 m1, Hs00161617 m1, and Hs00299807 m1 (Applied Biosystems, Darmstadt, Germany), respectively. mRNAs analyzed and primer sequences (confirmed by NCBI BLAST analysis) are listed in Table 2.

### 4.5. Western Blotting

A total of 60 h after transfection, both SKOV3 and CAOV3 cells were serum-starved for 12 h, before stimulation with 2 ng/mL recombinant human Midkine (PeproTech GmbH, Hamburg, Germany) for 0, 10, or 30 min, respectively. Cells were lysed after 72 h with 200 µL/well of RIPA-Buffer supplemented with a proteinase inhibitor cocktail containing NaF, β-glycerol phosphate, and NaVO_3_. BCA assay (Pierce, Schwerte, Germany) was used to determine the protein concentration of cell lysate. Samples were separated by SDS-polyacrylamide gel electrophoresis under reducing conditions and subsequently transferred to a nitrocellulose membrane. The membrane was blocked with 5% skim milk in TBST buffer, pH 7.6. The primary antibodies (*p*-STAT3^(Y705)^, rabbit monoclonal, 1:1000 Cell Signaling, Billerica, MA, USA; STAT3, rabbit monoclonal, 1:1000, Cell Signaling, Billerica, MA, USA; and GAPDH, monoclonal, mouse, 1:5000, Santa Cruz Biotechnologies, Dallas, TX, USA) were diluted in 5% BSA in TBST buffer and incubated with the membranes overnight at 4 °C. Membranes were first probed with p-STAT3 antibody and reprobed with the additional antibodies after stripping with 0.2 M glycine, pH 2.5, using the procedure indicated above. Following primary antibody incubation, the membranes were washed 3× with TBST and subsequently incubated with the horseradish peroxidase (HRP)-conjugated secondary goat anti-rabbit or goat anti-mouse antibodies (1:5000, Calbiochem, Darmstadt, Germany) in blocking solution (5% skim milk) for 1 h at room temperature. Membranes were subjected to chemiluminescence ECL reaction with SuperSignal™West Pico PLUS Chemiluminescent Substrate (Thermo Scientific™, cat. no. 34580, Foster City, CA, USA) and detected with a FUSION SL (Vilber Lourmat, Marne-la-Vallée Cedex, France) device.

### 4.6. Flow Cytometry

Flow cytometric detection of Syndecan-3 was performed 72 h after siRNA transfection. Both cell lines were washed twice with ice-cold PBS and harvested with 2 mM EDTA (AppliChem, cat. no. 141669.1211, Darmstadt, Germany) applied for 13 min at 37 °C. Cells were washed twice with PBS and resuspended in PBS containing 10% BSA before staining with anti-Syndecan-3 goat IgG APC-conjugated antibody (R&D Systems, Minneapolis, MN, USA), or goat IgG APC-conjugated antibody as isotype control (R&D Systems, Minneapolis, MN, USA) for 15 min in the dark. Cells were analyzed using a CyFlow Space (Sysmex/Partec, Münster, Germany) flow cytometer equipped with a 25 mW 638 nm red laser diode and a 20 mW 488 nm blue laser. The fluorescence was detected in FL5 at 675 nm (BP 675/30 nm). Fluorescence intensity was calculated as mean fluorescence intensity (MFI) by setting a region gate.

### 4.7. MTT Cell Viability and Chemosensitivity Assay

After 24 h post-transfection, 2000 transfected and control SKOV3 or CAOV3 cells were seeded in 96-well plates and cultured for 24 h, followed by 72 h incubation in the presence of a 1:2 dilution series of paclitaxel starting at 1000 nM and cisplatin at 8.3 µM, or a combination of 0–1000 nM paclitaxel and 0–114 nM cisplatin (ratio of 9 to 1) for SKOV3; 1:2 dilution series of paclitaxel starting at 160 nM and cisplatin at 3 µM or combinatorial treatment (9:1 paclitaxel/cisplatin (0–1000 nM paclitaxel, 0–3 µM cisplatin) for CAOV3 cells and 3-(4,5-dimethylthiazol-2-yl)-2,5-diphenyltetrazolium bromide (MTT to analyze cell viability and chemosensitivity). The ratio of paclitaxel and cisplatin is based on a dose of 175 mg/m^2^ paclitaxel and 20 mg/m^2^ cisplatin which is used in clinical treatment (ratio of 8.75 to 1). To prepare the solution of the combination paclitaxel–cisplatin, 30 µL paclitaxel (a 100 uM solution) dissolved in 3 mL water was added to 45 µL cisplatin (an 83 nM solution) dissolved in 3 mL water. The chemotherapeutics were applied by serial 1:2 dilution of the highest indicated concentration. After lysis, optical density measurement was performed at 595 nm in a microplate reader. The IC_50_ for each drug and treatment as well as the combination index can be found in Table 1.

### 4.8. Scratch Wound Assay

Scratch wound assays were performed to assess changes in cell motility. A total of 350,000 SKOV3 cells per well were cultivated in a six-well plate and left until they reached 100% confluence. After that, confluent SKOV3 cell monolayers were wounded by scraping once horizontally and vertically with a 100 µL pipette tip. The closing of the cell-depleted area was monitored by Nomarski contrast light microscopy and documented with a Zeiss Axiophot camera (Zeiss, Jena, Germany) immediately (t0) and 24 h after wounding (t24). NIH Image J software (NIH, Bethesda, MA, USA) was used to quantify the cell-free wounded area. Data are shown as percentage of the cell-free area of SDC3-silenced cells compared with controls.

### 4.9. Hanging Drop Assay for 3D Sphere Formation

Spheroids were generated using the hanging drop method as described [24]. SKOV3 and CAOV3 cells were harvested by trypsinization, washed, and resuspended in culture medium. A total of 10 drops with 20 µL of medium, each containing 10,000 cells per condition, were deposited on the top lid of a plastic Petri dish and the bottom chamber was filled with PBS (Sigma-Aldrich, cat. no. D1408). After 4 days and 7 days, the Petri dish lids were inverted and images of the 10 3D spheres per condition that were cultivated were taken with a Zeiss Axiophot (Zeiss, Jena, Germany) bright-field microscope (magnification 10×). The spheroid area was quantified with the NIH ImageJ program (U.S. National Institutes of Health, Bethesda, MD, USA).

### 4.10. Cell Colony Formation 

First, cells were transfected as described above. Then, 24 h after transfection, 35 × 10 mm tissue culture Petri dishes with vented lid, 2 × 2 mm grid, and a culture area of 8.8 cm (Nunc, Langenselbold, Germany) were used and 500 and 1000 SKOV3 and CAOV3 cells were seeded and then incubated for 10 days. A colony was defined as a contiguous cell group of more than 50 cells. Plating efficiency (PEf) was determined as PEf = colony number/number of seeded cells. Surviving fractions (SFs) of transfected cells were calculated relative to non-transfected controls (SF = PEf(transfected)/PEf(control)).

### 4.11. TNM, Kaplan–Meier Plot and STRING Analysis

The TNMplot database (https://www.tnmplot.com/, accessed on 4 June 2021) [18] integrates public gene expression data of ovarian cancer specimens, ovarian cancer metastases and normal tissue from the GEO, GTEx and TCGA datasets. mRNA expression of SDC3 in these categories was independently assessed using the RNASeq dataset for comparing ovarian serous cystadenocarcinoma tissue (*n* = 374) compared with control tissue (*n* = 133), and the Affymetrix-based gene array dataset comparing ovarian cancer tissue (*n* = 744), ovarian cancer metastases (*n* = 44), and normal tissue (*n* = 46). For the survival analysis, the publicly available gene expression database Kaplan–Meier plotter (KM plotter) (http://kmplot.com/analysis/index.php?p=service&cancer=ovar, accessed on 4 June 2021) was used [27]. This database integrates gene expression data and survival information of 1435 ovarian cancer patients downloaded from the public repository Gene Expression Omnibus (GEO). We analyzed the expression of SDC3 and visualized its correlation to relapse-free survival by generating Kaplan–Meier survival plots. High and low expression of SDC3 was determined using the default cut-off (462; expression range of the probe:6–2764), the Jet-set best probe set, and a follow-up of 180 months. The Affymetrix probe set ID for SDC3 is 202898_at. The online platform STRING (https://string-db.org, accessed on 4 June 2021) [30] was used to develop the in silico protein interaction networks for SDC3, testing first shell interactions of SDC3 with the differentially regulated gene products identified in this study. All interactions were predicted with a medium confidence threshold of 0.400.

### 4.12. Microarray Gene Expression Database of Epithelial Ovarian Cancer Subtype CSIOVDB

To corroborate the survival data of ovarian cancer patients obtained with kmplot, we use the online platform CSIOVDB (http://csiovdb.mc.ntu.edu.tw/CSIOVDB.html, retrieved on 26 March 2022) to analyze the expression of SDC3 and correlates with the survival of ovarian cancer patients. CSIOVDB transcriptomic database which contains 3431 human ovarian cancers, and also comprises stroma and epithelium from normal ovary tissue. In this database, the molecular subtype and epithelial–mesenchymal transition status for each ovarian cancer sample, with major ovarian cancer histologies can be analyzed as well as the clinicopathological parameters such as tumor grade, surgical debulking status, clinical response, and age. The overall and disease-free survival rates from 1868 and 1516 samples can be analyzed [28].

### 4.13. Statistical Analysis

For statistical analysis, 95% confidence intervals (CI) were determined by calculating arithmetic mean values and variance (standard deviation, SD or standard error of the mean SEM) of at least three independent experiments. To evaluate whether differences between the values obtained were significant, the Mann–Whitney U test was used to assess the effect of SDC3 knockdown vs. control siRNA in all in vitro data using IBM SPSS Statistics software, version 27 (IBM, Armonk, NY, USA). A *p*-value < 0.05 was considered statistically significant. For TNMPlot data, statistical analysis in the RNASeq dataset was performed using the Mann–Whitney U test, whereas the gene array dataset samples were analyzed by the Kruskal–Wallis test using Dunn’s test for post-hoc group comparisons. Statistical analysis of the KM Plotter dataset was performed using the package ‘survival’ to calculate and plot Kaplan–Meier survival curves. Hazard ratio (HR; and 95% confidence intervals) and log rank *p*-values are calculated and displayed in the output files.

## Figures and Tables

**Table 1:** IC50 for Taxol and cisplatin, and combination index (CI) for the combination of both drugs in SKOV3 and CAOV3 cells. CI < 1 indicates synergistic effect, CI = 1 indicates additive effects and CI > 1 indicates antagonistic effect.

Cell Line	Drug	IC_50_ [nM]			Combination Index (CI) [nM]		
		Control	Sequence 1	Sequence 2	Control	Sequence 1	Sequence 2
SKOV3	Taxol	2.909	1.679	0.4900	-	-	-
	Cisplatin	2.270	2.887	1.355	-	-	-
	Taxol/Cisplatin	-	-	-	0.930	18.198	<0.001
CAOV3	Taxol	1.952	1.947	1.985	-	-	-
	Cisplatin	1.222	1.304	1.360		-	-
	Taxol/Cisplatin	-	-	-	13.013	10.649	12.774

**Table 2:** PCR primer sequences used in this study.

mRNA	Sequence (Forward)	Sequence (Reverse)
ACTB	TCAAGATCATTGCTCCTCCTGAG	ACATCTGCTGGAAGGTGGACA
CDH1	CAAAGCCCAGAATCCCCAAG	CACACCTGGAATTGGGCAAA
Gli1	TTCCTACCAGAGTCCCAAGT	CCCTATGTGAAGCCCTATTT
Gli2	GTCAGAGCCATCAAGACCGAGA	GCATCTCCACGCCACTGTCATT
Gli3	TCAGCAAGTGGCTCCTATGGTC	GCTCTGTTGTCGGCTTAGGATC
Hes1	GTGAAGCACCTCCGGAAC	CGTTCATGCACTCGCTGA
Hey2	TTGCCCATGCCTAAACTAGTGC	TCTCACGTGCTTGATTTCAGCATA
Msi1	AGCTTCCCTCTCCCTCATTCC	GGAATTCCAGGGTCCTGAGC
Msi2	CCTAGTATGCTTGCCTCACAAACG	CGTCATCAGGGAGAAGCACAG
Notch1	GGTGAGACCTGCCTGAATG	GTTGGGGTCCTGGCATC
PTCH1	GGTGGCACAGTCAAGAACA	ACCAAGAGCGAGAAATGG
Robo1	GCATCGCTGGAAGTAGCCATACT	CATGAAATGGTGGGCTCAGGAT
SDC3	TGCCTCAGAAGAGTATCCTG	CTTGTCAGGCTTCTGGTATG
SFRP1	GATGCAGGAGGCTCAGGTGAT	GCTGGCAACAGGTCAGAACG
SHH	GAAAGCAGAGAACTCGGTGG	GGAAAGTGAGGAAGTCGCTG
Snail	CGAGCCCAGGCAGCTATTTC	CCCGACAAGTGACAGCCATT
Src	ACCACCTTTGTGGCCCTCTATG	GCCACCAGTCTCCCTCTGTGTT
TCF7L1	AAGGTGCCTGCCACTTCCTC	CCTGCCACTCTGGGATTGTG
TCF7L2	AAACCAGCTGCCGCTTTTATG	GCAACATCAACATGCCTAGGTTTT
Twist	GCGGCCAGGTACATCGACTT	TGCAGCTTGCCATCTTGGAG
Vimentin	TCAGCATCACGATGACCTTGAA	CTGCAGAAAGGCACTTGAAAGC
Wnt 3A	AGGTCCCACAGCCCTGAGAT	TCCAGGAAAGCGGACCATTT
Wnt 5A	TCGTTAGCAGCATCAGTCCACA	GACCTGTGCCTTCGTGCCTA
ZEB1	CAGCCCTGCAGTCCAAGAAC	TTGTCTTTCATCCTGATTTCCATTT

## Data Availability

All data presented in this study are available in the manuscript and supplementary files.

## Supplementary Materials

The following are available online at https://www.mdpi.com/article/10.3390/ijms23105793/s1.
