# Supplementary figures and images for: The Cell Surface Heparan Sulfate Proteoglycan Syndecan-3 Promotes Ovarian Cancer Pathogenesis
Source: Int J Mol Sci. 2022 May 21;23(10):5793. doi: 10.3390/ijms23105793 (PMC9145288; doi:10.3390/ijms23105793)

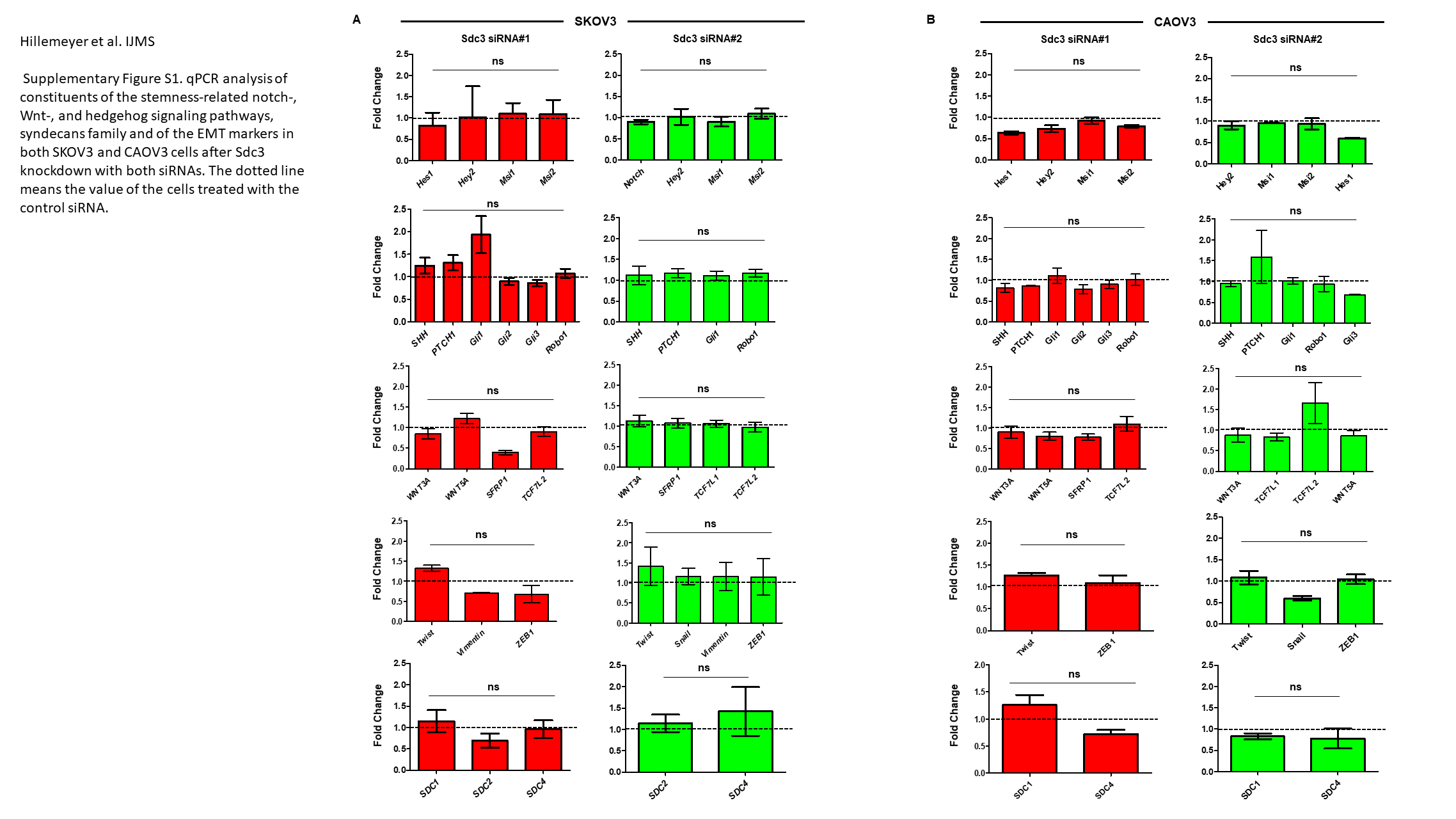

Supplement: Supplementary file 1 [file ijms-23-05793-s001.zip › Figure S1.TIF]

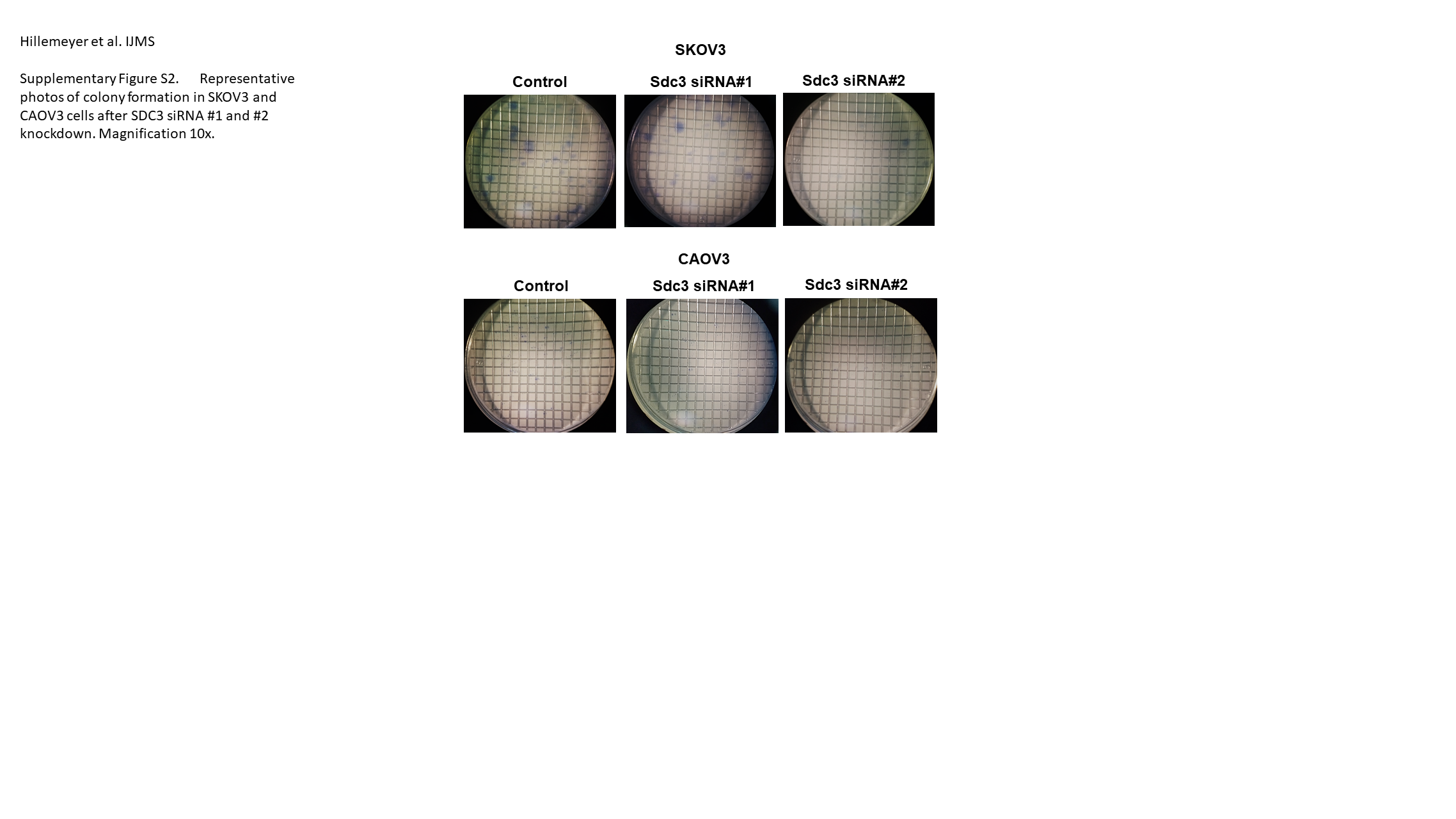

Supplement: Supplementary file 1 [file ijms-23-05793-s001.zip › Figure S2.TIF]
